# KLK7 expression in human tumors: a tissue microarray study on 13,447 tumors

**DOI:** 10.1186/s12885-024-12552-8

**Published:** 2024-07-03

**Authors:** Simon Kind, Carolina Palacios Castillo, Ria Schlichter, Natalia Gorbokon, Maximilian Lennartz, Lisa S. Hornsteiner, Sebastian Dwertmann Rico, Viktor Reiswich, Florian Viehweger, Martina Kluth, Claudia Hube-Magg, Christian Bernreuther, Franziska Büscheck, Till S. Clauditz, Christoph Fraune, Andrea Hinsch, Till Krech, Patrick Lebok, Stefan Steurer, Eike Burandt, Sarah Minner, Andreas H. Marx, Ronald Simon, Waldemar Wilczak, Guido Sauter, Anne Menz, Frank Jacobsen

**Affiliations:** 1 Institute of Pathology, University Medical Center Hamburg-Eppendorf, Martinistr. 52, Hamburg, 20246 Germany; 2 Institute of Pathology, Clinical Center Osnabrueck, Osnabrueck, Germany; 3 Department of Pathology, Academic Hospital Fuerth, Fuerth, Germany

**Keywords:** KLK7, Tissue microarray, Immunohistochemistry, Neoplastic human tissues

## Abstract

**Background:**

Kallikrein-related peptidase 7 (KLK7) is a chymotrypsin-like serine protease which is essential for the desquamation of corneocytes and thus plays a pivotal role in maintaining skin homeostasis. In cancer, KLK7 overexpression was suggested to represent a route for metastasis through cleavage of cell junction and extracellular matrix proteins of cancer cells.

**Methods:**

To comprehensively determine KLK7 protein expression in normal and neoplastic tissues, a tissue microarray containing 13,447 samples from 147 different tumor types and subtypes as well as 608 samples of 76 different normal tissue types was analyzed by immunohistochemistry.

**Results:**

KLK7 positivity was found in 64 of 147 tumor categories, including 17 tumor categories with at least one strongly positive case. The highest rate of KLK7 positivity was found in squamous cell carcinomas from various sites of origin (positive in 18.1%-63.8%), ovarian and endometrium cancers (4.8%-56.2%), salivary gland tumors (4.8%-13.7%), bilio-pancreatic adenocarcinomas (20.0%-40.4%), and adenocarcinomas of the upper gastrointestinal tract (3.3%-12.5%). KLK7 positivity was linked to nodal metastasis (*p* = 0.0005), blood vessel infiltration (*p* = 0.0037), and lymph vessel infiltration (*p* < 0.0001) in colorectal adenocarcinoma, nodal metastasis in hepatocellular carcinoma (*p* = 0.0382), advanced pathological tumor stage in papillary thyroid cancer (*p* = 0.0132), and low grade of malignancy in a cohort of 719 squamous cell carcinomas from 11 different sites of origin (*p* < 0.0001).

**Conclusions:**

These data provide a comprehensive overview on KLK7 expression in normal and neoplastic human tissues. The prognostic relevance of KLK7 expression and the possible role of KLK7 as a drug target need to be further investigated.

**Supplementary Information:**

The online version contains supplementary material available at 10.1186/s12885-024-12552-8.

## Introduction

The human kallikrein (KLK, kallikrein-related peptidase) family refers to a group of enzymes that belong to the proteolytic family of serine proteases. To date, there are 15 known members in the human kallikrein family, designated as KLK1-KLK15 [1]. Some kallikreins have been implicated in cancer. For example, KLK3 (prostate specific antigen, PSA) is used as a blood biomarker for prostate cancer [2], and alterations of KLK4, KLK5, and KLK6 have been suggested to play a role for breast cancer aggressiveness [3]. A cancer-relevant role is also suspected for Kallikrein-related peptidase 7. KLK7 is a chymotrypsin-like serine protease with a critical role in the maintenance of skin homeostasis [4–6]. The KLK7 zymogen is secreted by a subset of keratinocytes into the stratum granulosum of the epidermis where it is activated by proteolytic removal of the short N-terminal [4, 7]. Activated KLK7 induces the desquamation of corneocytes from the outer layer of the keratinized squamous epithelium of the epidermis by proteolysis of corneodesmosomes [5, 8]. Dysregulated KLK7 production and secretion occurs in various skin disorders going along with dry, crusty and inflamed skin such as atopic dermatitis, Netherton syndrome, or psoriasis (summarized in [9]).

In normal tissues, KLK7 expression is largely limited to squamous epithelium [4, 5]. In cancer, however, KLK7 expression has been described to occur in a broad range of tumor entities [10–27]. Overexpression of KLK7 has been suggested to promote epithelial-mesenchymal transition (EMT) through the hydrolysis of cell membrane and extracellular matrix molecules (e.g., E-cadherin) and therefore to represent a possible mechanism for metastasis (summarized in [28]). Several studies have described high rates of KLK7 positivity in triple negative breast cancer [17], colorectal cancer [21], ovarian cancer [12–14], adenocarcinomas of the cervix uteri [23, 24], pancreatic cancer [10, 11] and malignant melanoma [19], but the published data on the prevalence of KLK7 immunostaining are discrepant for several tumor entities. For example, KLK7 positivity has been found in 20.0%-92.5% of adenocarcinomas of the endocervix [18, 23, 24] and from 41.0%–100% of squamous cell carcinomas of the oral cavity [20, 22, 25, 26]. Many important tumor entities such as carcinomas of the lung, stomach, esophagus, liver, bile ducts, or the endometrium have not been analyzed for KLK7 expression yet.

To better understand the prevalence and potential role of KLK7 expression in cancer, a comprehensive study analyzing large numbers of neoplastic and non-neoplastic tissues under highly standardized conditions is desirable. For this purpose, KLK7 expression was analyzed in more than 13,000 tumor tissue samples from 147 different tumor types and subtypes as well as 76 non-neoplastic tissue categories by immunohistochemistry (IHC) in a tissue microarray (TMA) format in this study.

## Materials and methods

### Tissue Microarrays (TMAs)

The tissue microarrays that were utilized in the current study had been described before [29–31]. In brief, our normal tissue TMA contains 76 different normal tissue types, and each tissue is represented by 8 samples from 8 different donors (in total: 608 samples on one slide). Our cancer TMAs were constructed from a total of 13,447 primary tumors obtained from 147 tumor types and subtypes that were distributed across 24 TMA slides. Detailed histopathological data on grade, pathological tumor stage (pT), pathological lymph node status (pN), blood vessel infiltration (V), and lymph vessel infiltration (L) were available from subsets of colon carcinomas (*n* = 2,351), liver carcinomas (*n* = 301), endometrium carcinomas (*n* = 259), ovarian carcinomas (*n* = 524), pancreas carcinomas (*n* = 598), papillary thyroid carcinomas (*n* = 382), gastric carcinomas (*n* = 398), as well as of 902 squamous cell carcinomas of different sites of origin. All samples were collected from the Institute of Pathology, University Hospital of Hamburg, Germany, the Institute of Pathology, Clinical Center Osnabrueck, Germany, and the Department of Pathology, Academic Hospital Fuerth, Germany. All tissues were formalin-fixed (4% buffered formalin) and subsequently embedded in paraffin. The process of TMA construction has been described earlier [32, 33]. In brief, each tissue is represented by a single 0.6 mm tissue core that was taken from a cancer containing donor block and transferred into an empty recipient paraffin block. The local ethics committee (Ethics commission Hamburg, WF-049/09) has approved the manufacturing and analysis of TMAs from archived remnants of diagnostic tissues. Patient informed consent was not required for this study. Patient data analysis without informed patient consent is covered by local laws (HmbKHG, §12). All work has been carried out in compliance with the Helsinki Declaration.

### Immunohistochemistry (IHC)

Freshly cut tissue sections were used for all IHC experiments. All immunohistochemistry experiments were made manually in one day at the Institute of Pathology, University Medical Center Hamburg-Eppendorf. TMA sections of 3µ were cut, deparaffinized in xylol and rehydrated through a descending alcohol series. Immunohistochemistry was performed manually. Sections were placed for 5 min in an autoclave at 121 °C in pH 7.8 DakoTarget Retrieval Solution™ (Agilent, CA, USA) for heat induced antigen retrieval. Endogenous peroxidase was blocked in Dako Peroxidase Blocking Solution™ (Agilent, CA, USA; #52,023). Primary antibody specific for KLK7 protein (mouse monoclonal, MSVA-707M, #4860-707M, MS Validated Antibodies, Hamburg, Germany) was applied at 37 °C for 60 min at a dilution of 1:150. Specificity and cross reactivity of MSVA-707M was checked by Western blotting (Supplementary Fig. 1) and by comparison of the staining pattern with that of a second independent antibody as recommended by The International Working Group for Antibody Validation (IWGAV) [34]. For this purpose, the normal tissue TMA was also analyzed by the rabbit recombinant monoclonal KLK7 antibody EPR22594-203 (Abcam; ab254258) at a dilution of 1:7.5 and an otherwise identical protocol. As controls, skin with stratum granulosum (positive control) and colon mucosa (negative control) were included in the TMA. The EnVision detection Kit™ (Agilent, CA, USA; #K5007) was used to visualize the bound antibody. For counterstain, slides were immersed in haemalaun solution. Staining was membranous and/or cytoplasmic, which fits to the function of KLK7 as a protein that is secreted from the cytoplasm via the cell membrane to the extracellular space [28]. Accordingly, cytoplasmic and membranous staining was scored. Slide scoring was performed as described before [29–31]. In brief, one pathologist scored all slides (SK). In questionable cases, a second pathologist’s opinion was sought. For scoring of tumor tissues, the percentage of KLK7 positive tumor cells was estimated in each spot and the staining intensity was noted in a 4-step scale (0, 1 + , 2 + , 3 +), where 0 indicates no visble staining, 1 + ligth brown staining, 3 + dark brown staining, and 2 + staining between 1 + and 3 + . Examples for the staining intensities are given in Supplementary Fig. 2. The staining results were combined into four groups for statistical analyses: Negative staining: no visible staining, weak staining: staining intensity of 1 + in ≤ 70% or staining intensity of 2 + in ≤ 30% of tumor cells, moderate staining: staining intensity of 1 + in > 70%, staining intensity of 2 + in > 30% but in ≤ 70% or staining intensity of 3 + in ≤ 30% of tumor cells, strong staining: staining intensity of 2 + in > 70% or staining intensity of 3 + in > 30% of tumor cells.

### KLK7 RNA expression data source

RNA expression z-scores of KLK7 relative to diploid samples from RNA sequencing (RNA-Seq by Expectation–Maximization (RSEM) V2, normalized from Illumina HiSeq) were downloaded from cBioportal (http://www.cbioportal.org) in July 2023. The data selection included the 32 studies labeled as “TCGA PanCancer Atlas Studies” with a total of 10,976 samples.

### Western Blot

Human tissue samples of skin, kidney and liver were shock-frozen in liquid nitrogen, grinded and lysed with 5% SDS. Samples were heated for 5 min at 95 °C and the suspension was centrifuged at 10,000 g for 5 min at 8 °C. Supernatant was further used for protein quantification via Qubit protein quantification kit (Invitrogen, Carlsbad, California, USA). Protein concentration was adjusted with sample buffer (4XLDS, BioRad, Hercules, California, USA) and DTT (1:10) and the sample was heated up to 95 °C for 5 min. 30 µg per human tissue sample was used for separation on a Bis–Tris 4–20% gel. The gel was then blotted on a 0.2 µm nitrocellulose membrane. The membrane was blocked for 1 h in 5% BSA in PBST before the primary murine antibody KLK7 (MSVA-707M, MS Validated Antibodies, GmbH, Hamburg, Germany, diluted 1:1,000 in 5% BSA in PBST) was incubated on the membrane over night at 4 °C. On the next day, the membrane was incubated with the secondary antibody goat-anti-mouse-HRP (1:2,000) for 1 h at room temperature and developed with Pierce ECL Western Blotting Substrate (BioRad). Blots were imaged using a BioRad Chemidoc imager. For loading control, the membrane was incubated with a primary anti-tubulin antibody (anti-rabbit, Abcam, Cambridge, UK, ab4074) using the same protocol.

### Statistics

The JMP 16 software package (SAS Institute Inc., NC, USA) was used for statistical analysis. Contingency table analysis and chi^2^-testing was applied to search for associations between KLK7 immunostaining and tumor phenotype. For statistical adjustment the Bonferroni correction (*p* = *p*-value/number of statistical tests) was performed, and *p* ≤ 0.0018 was considered as statistically significant.

## Results

### Technical issues

A total of 12,345 (91.8%) of 13,447 tumor samples and at least 4 normal samples per tissue category were interpretable in our TMA analysis. Non-interpretable samples demonstrated lack of unequivocal tumor cells or absence of tissue in the respective TMA spots.

### KLK7 in normal tissues

**Fig. 1:**
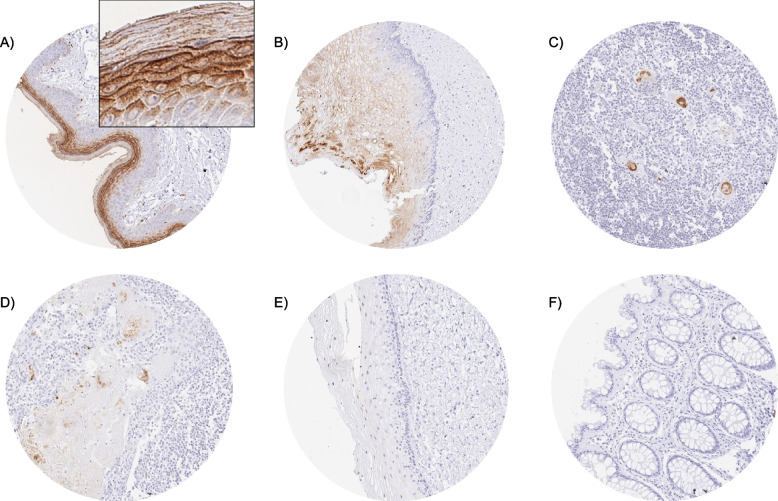
KLK7 immunostaining in normal tissues. The panels show a strong membranous and cytoplasmic KLK7 staining of the granular layer of the epidermis of the skin (**A**) (inset shows higher magnification of the granular layer), a weak to moderate KLK7 positivity of the upper two thirds of the squamous epithelium of the esophagus (**B**), a strong KLK7 staining around the keratinizing zone of corpuscles of Hassall‘s of the thymus (**C**), and a weak to moderate KLK7 positivity in a subset of cells of squamous epithelium of tonsil crypts (**D**). KLK7 staining is largely absent in non-keratinizing squamous epithelium of the uterine cervix (**E**) and completely lacking in the mucosa of the colon (**F**)

KLK7 immunostaining was predominantly seen in squamous epithelia where the staining preferably occurred in a zone between the middle and the top 20% of the squamous epithelium. This area contains the granular layer in keratinizing squamous epithelium. Staining was predominantly membranous but in case of high staining intensity the cytoplasm was also involved. The extent of KLK7 staining in squamous epithelium also depended on its localization. KLK7 staining was low in the cervix uteri, higher in the esophagus and tonsil surface, and highest in the skin. In the skin, a strong staining was seen in the stratum granulosum and the keratinizing cell layers. KLK7 immunostaining was also strong in the cortex of hair follicles, the luminal area of sebaceous glands, and the central (keratinizing) zone of corpuscles of Hassall’s in the thymus. Few scattered epithelial cells or groups of cells with a moderate KLK7 positivity were occasionally seen in renal tubuli, the fallopian tube, and in salivary glands. Representative images of KLK7 positive normal tissues are shown in Fig. 1. All these findings were observed by both MSVA-707M and EPR22594-203 (Supplementary Fig. 3). An additional cytoplasmic smooth muscle staining and nuclear staining was observed in several organs for EPR22594-203 but not for MSVA-707M and was thus considered an antibody specific cross-reactivity of EPR22594-203.

### KLK7 in cancer tissues

**Fig. 2:**
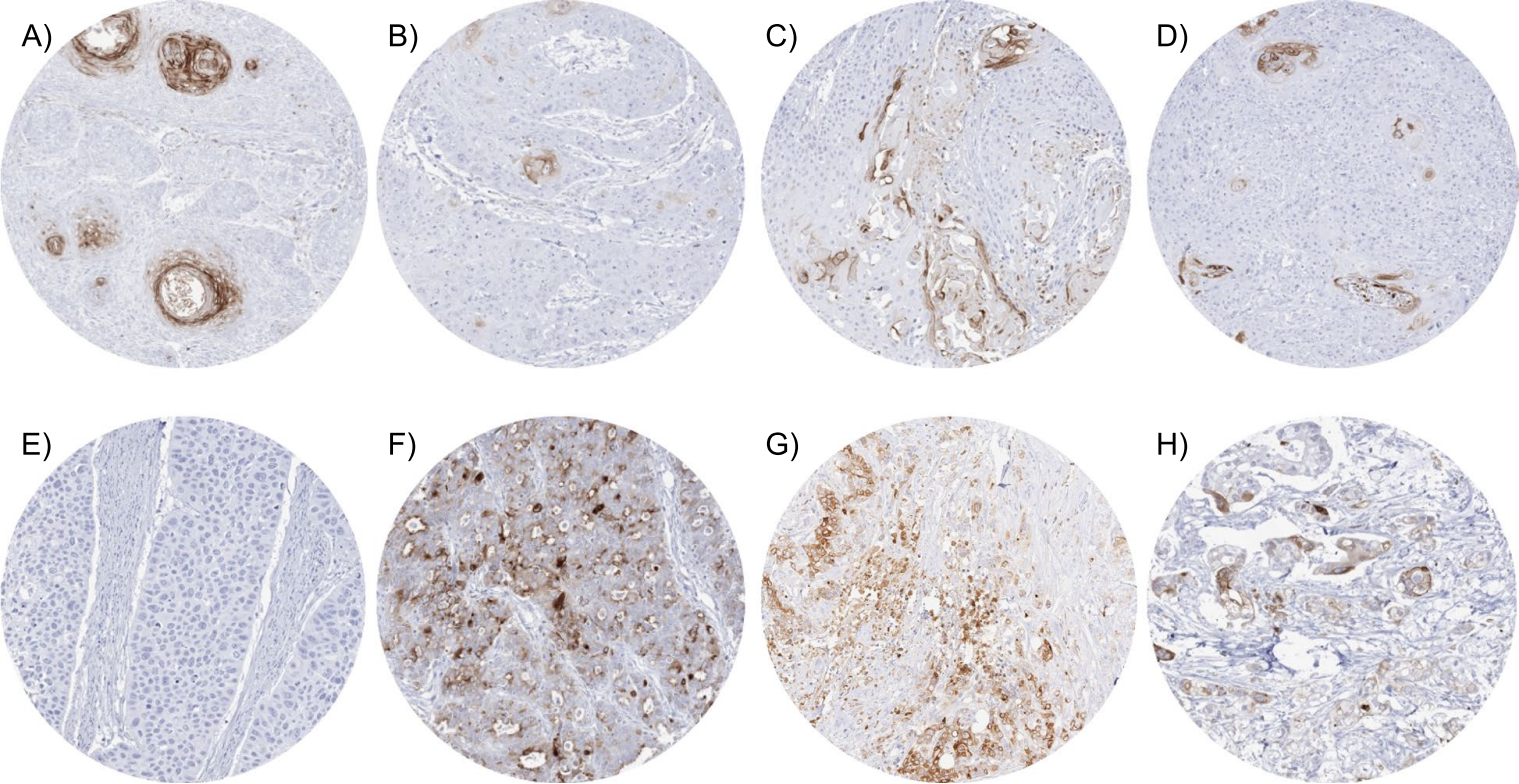
KLK7 immunostaining in cancer. The panels show a focal KLK7 immunostaining (staining intensity (int) 2 + in 10% of tumor cells) in areas of keratinization of an HPV-negative squamous cell carcinoma of the vulva (**A**), the oral cavity (int 1 + in 10%) (**B**), and of the urinary bladder (int 1 + in 20%) (**C**). A focal KLK7 staining is also seen in a urothelial carcinoma of the bladder with focal squamous differentiation (int 2 + in 10%) (**D**) while KLK7 staining is absent in a poorly differentiated squamous cell carcinoma of the esophagus lacking keratinization (int 0) (**E**). KLK7 positivity of variable intensity is also seen in samples from a serous high-grade carcinoma of the ovary (int 2 + in 40%) (**F**) as well as from an intestinal adenocarcinoma of the stomach (int 2 + in 30%) (**G**) and the pancreas (int 2 + in 30%) (**H**)

**Fig. 3:**
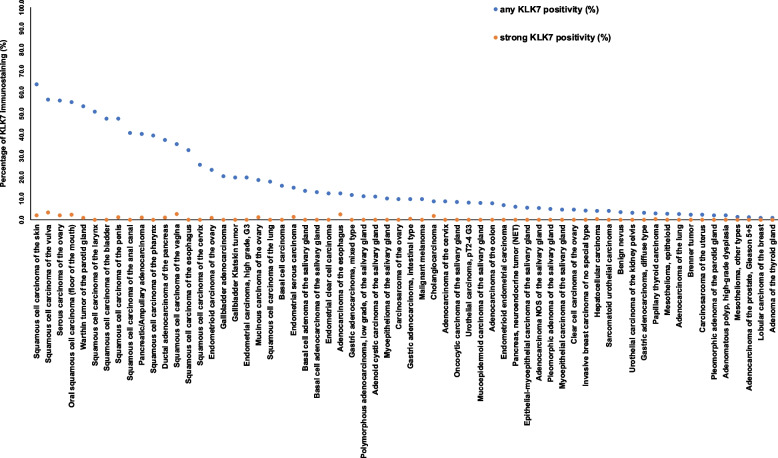
Ranking order of KLK7 immunostaining in cancers. Both the percentage of positive cases (blue dots) and the percentage of strongly positive cases (orange dots) are shown

Positive KLK7 immunostaining was detectable in 1,600 (13.0%) of the 12,345 analyzable tumors, including 1,352 (11.0%) with weak, 203 (1.6%) with moderate, and 45 (0.4%) with strong immunostaining. Overall, 64 (43.5%) of 147 tumor categories showed detectable KLK7 expression with 17 (11.6%) tumor categories including at least one case with strong positivity (Table 1). Representative images of KLK7 positive tumors are shown in Fig. 2. The highest rate of KLK7 positivity was found in squamous cell carcinomas from various sites of origin (positive in 18.1%-63.8%), various categories of ovarian and endometrium cancers (4.8%-56.2%), salivary gland tumors (4.8%-13.7%), bilio-pancreatic adenocarcinomas (20.0%-40.4%), and adenocarcinomas of the upper gastrointestinal tract (3.3%-12.5%). Twenty other tumor entities showed—a usually weak—KLK7 positivity in less than 9%. A graphical representation of a ranking order of KLK7 positive and strongly positive cancers is given in Fig. 3. The relationship between KLK7 expression and clinically important histopathological and molecular tumor features in colon, liver, endometrial, ovarian, pancreas, papillary thyroid, and gastric carcinomas is shown in Table 2. Detectable KLK7 immunostaining was linked to pN + (*p* = 0.0005), V1 (*p* = 0.0037), and L1 (*p* < 0.0001) in colorectal adenocarcinoma, pN + in hepatocellular carcinoma (*p* = 0.0382), advanced pT stage in papillary thyroid cancer (*p* = 0.0132), and low grade of malignancy in a cohort of 719 squamous cell carcinomas from 11 different sites of origin (*p* < 0.0001).

**Table 1:** KLK7 immunostaining in human tumors

	**Tumor entity**		**KLK7 immunostaining result**				
		on TMA (*n*)	analyzable (*n*)	negative (%)	weak (%)	moderate (%)	strong (%)
**Tumors of the skin**	Pilomatrixoma	35	23	100.0	0.0	0.0	0.0
	Basal cell carcinoma	89	75	84.0	9.3	6.7	0.0
	Benign nevus	29	28	96.4	0.0	3.6	0.0
	Squamous cell carcinoma of the skin	145	141	36.2	55.3	6.4	2.1
	Malignant melanoma	65	62	90.3	8.1	1.6	0.0
	Malignant melanoma Lymph node metastasis	86	79	100.0	0.0	0.0	0.0
	Merkel cell carcinoma	48	43	100.0	0.0	0.0	0.0
**Tumors of the head and neck**	Squamous cell carcinoma of the larynx	109	106	49.1	48.1	2.8	0.0
	Squamous cell carcinoma of the pharynx	60	58	60.3	36.2	3.4	0.0
	Oral squamous cell carcinoma (floor of the mouth)	130	128	44.5	46.1	7.0	2.3
	Pleomorphic adenoma of the parotid gland	50	47	97.9	0.0	2.1	0.0
	Warthin tumor of the parotid gland	104	103	46.6	38.8	13.6	1.0
	Adenocarcinoma, NOS (Papillary Cystadenocarcinoma)	14	12	100.0	0.0	0.0	0.0
	Salivary duct carcinoma	15	14	100.0	0.0	0.0	0.0
	Acinic cell carcinoma of the salivary gland	181	160	100.0	0.0	0.0	0.0
	Adenocarcinoma NOS of the salivary gland	109	89	94.4	5.6	0.0	0.0
	Adenoid cystic carcinoma of the salivary gland	180	118	89.0	11.0	0.0	0.0
	Basal cell adenoma of the salivary gland	101	95	86.3	13.7	0.0	0.0
	Basal cell adenocarcinoma of the salivary gland	25	23	87.0	13.0	0.0	0.0
	Epithelial-myoepithelial carcinoma of the salivary gland	53	53	94.3	5.7	0.0	0.0
	Mucoepidermoid carcinoma of the salivary gland	343	300	92.0	7.7	0.3	0.0
	Myoepithelial carcinoma of the salivary gland	21	21	95.2	4.8	0.0	0.0
	Myoepithelioma of the salivary gland	11	10	90.0	10.0	0.0	0.0
	Oncocytic carcinoma of the salivary gland	12	12	91.7	8.3	0.0	0.0
	Polymorphous adenocarcinoma, low grade, of the salivary gland	41	36	88.9	8.3	2.8	0.0
	Pleomorphic adenoma of the salivary gland	53	39	94.9	2.6	2.6	0.0
**Tumors of the lung, pleura and thymus**	Adenocarcinoma of the lung	196	187	97.3	2.7	0.0	0.0
	Squamous cell carcinoma of the lung	80	72	81.9	16.7	1.4	0.0
	Small cell carcinoma of the lung	16	16	100.0	0.0	0.0	0.0
	Mesothelioma, epitheloid	40	35	97.1	2.9	0.0	0.0
	Mesothelioma, other types	77	72	98.6	0.0	1.4	0.0
	Thymoma	29	28	100.0	0.0	0.0	0.0
**Tumors of the female genital tract**	Squamous cell carcinoma of the vagina	78	73	64.4	28.8	4.1	2.7
	Squamous cell carcinoma of the vulva	157	145	43.4	48.3	4.8	3.4
	Squamous cell carcinoma of the cervix	136	131	74.0	22.9	3.1	0.0
	Adenocarcinoma of the cervix	23	23	91.3	4.3	4.3	0.0
	Endometrioid endometrial carcinoma	338	289	93.1	6.6	0.3	0.0
	Endometrial serous carcinoma	86	73	84.9	12.3	1.4	1.4
	Carcinosarcoma of the uterus	57	42	97.6	2.4	0.0	0.0
	Endometrial carcinoma, high grade, G3	13	10	80.0	20.0	0.0	0.0
	Endometrial clear cell carcinoma	9	8	87.5	12.5	0.0	0.0
	Endometrioid carcinoma of the ovary	130	106	76.4	18.9	3.8	0.9
	Serous carcinoma of the ovary	580	527	43.8	45.5	8.5	2.1
	Mucinous carcinoma of the ovary	101	80	81.3	16.3	1.3	1.3
	Clear cell carcinoma of the ovary	51	42	95.2	2.4	0.0	2.4
	Carcinosarcoma of the ovary	47	41	90.2	9.8	0.0	0.0
	Granulosa cell tumor of the ovary	44	43	100.0	0.0	0.0	0.0
	Leydig cell tumor of the ovary	4	4	100.0	0.0	0.0	0.0
	Sertoli cell tumor of the ovary	1	1	100.0	0.0	0.0	0.0
	Sertoli Leydig cell tumor of the ovary	3	3	100.0	0.0	0.0	0.0
	Steroid cell tumor of the ovary	3	3	100.0	0.0	0.0	0.0
	Brenner tumor	41	41	97.6	2.4	0.0	0.0
**Tumors of the breast**	Invasive breast carcinoma of no special type	80	68	95.6	1.5	2.9	0.0
	Lobular carcinoma of the breast	122	108	99.1	0.9	0.0	0.0
	Medullary carcinoma of the breast	15	15	100.0	0.0	0.0	0.0
	Tubular carcinoma of the breast	18	18	100.0	0.0	0.0	0.0
	Mucinous carcinoma of the breast	22	20	100.0	0.0	0.0	0.0
	Phyllodes tumor of the breast	50	49	100.0	0.0	0.0	0.0
**Tumors of the digestive system**	Adenomatous polyp, low-grade dysplasia	50	50	100.0	0.0	0.0	0.0
	Adenomatous polyp, high-grade dysplasia	50	48	97.9	2.1	0.0	0.0
	Adenocarcinoma of the colon and rectum	2483	2354	92.3	7.2	0.5	0.0
	Gastric adenocarcinoma, diffuse type	215	209	96.7	2.9	0.5	0.0
	Gastric adenocarcinoma, intestinal type	215	205	90.2	8.8	0.5	0.5
	Gastric adenocarcinoma, mixed type	62	60	88.3	11.7	0.0	0.0
	Adenocarcinoma of the esophagus	83	80	87.5	10.0	0.0	2.5
	Squamous cell carcinoma of the esophagus	76	64	67.2	32.8	0.0	0.0
	Squamous cell carcinoma of the anal canal	91	88	59.1	35.2	5.7	0.0
	Cholangiocarcinoma	58	57	91.2	7.0	0.0	1.8
	Gallbladder adenocarcinoma	51	34	79.4	11.8	8.8	0.0
	Extrahepatic bile duct carcinoma	42	40	80.0	17.5	2.5	0.0
	Hepatocellular carcinoma	312	285	95.8	3.2	0.7	0.4
	Ductal adenocarcinoma of the pancreas	659	631	62.4	30.3	6.2	1.1
	Pancreatic/Ampullary adenocarcinoma	98	94	59.6	27.7	11.7	1.1
	Acinar cell carcinoma of the pancreas	16	15	100.0	0.0	0.0	0.0
	Gastrointestinal stromal tumor (GIST)	62	62	100.0	0.0	0.0	0.0
**Tumors of the urinary system**	Non-invasive papillary urothelial carcinoma, pTa G3	32	27	100.0	0.0	0.0	0.0
	Urothelial carcinoma, pT2-4 G3	162	136	91.9	6.6	1.5	0.0
	Squamous cell carcinoma of the bladder	22	21	52.4	47.6	0.0	0.0
	Small cell neuroendocrine carcinoma of the bladder	22	22	100.0	0.0	0.0	0.0
	Sarcomatoid urothelial carcinoma	25	24	95.8	0.0	4.2	0.0
	Urothelial carcinoma of the kidney pelvis	62	59	96.6	1.7	1.7	0.0
	Clear cell renal cell carcinoma	50	41	100.0	0.0	0.0	0.0
	Papillary renal cell carcinoma	50	31	100.0	0.0	0.0	0.0
	Chromophobe renal cell carcinoma	50	34	100.0	0.0	0.0	0.0
	Oncocytoma	50	30	100.0	0.0	0.0	0.0
**Tumors of the male genital organs**	Adenocarcinoma of the prostate, Gleason 3 + 3	83	83	100.0	0.0	0.0	0.0
	Adenocarcinoma of the prostate, Gleason 4 + 4	80	76	100.0	0.0	0.0	0.0
	Adenocarcinoma of the prostate, Gleason 5 + 5	85	82	98.8	1.2	0.0	0.0
	Adenocarcinoma of the prostate (recurrence)	258	246	100.0	0.0	0.0	0.0
	Small cell neuroendocrine carcinoma of the prostate	19	18	100.0	0.0	0.0	0.0
	Seminoma	111	93	100.0	0.0	0.0	0.0
	Embryonal carcinoma of the testis	54	46	100.0	0.0	0.0	0.0
	Leydig cell tumor of the testis	31	29	100.0	0.0	0.0	0.0
	Sertoli cell tumor of the testis	2	2	100.0	0.0	0.0	0.0
	Sex cord stromal tumor of the testis	1	1	100.0	0.0	0.0	0.0
	Spermatocytic tumor of the testis	1	1	100.0	0.0	0.0	0.0
	Yolk sac tumor	53	43	100.0	0.0	0.0	0.0
	Teratoma	53	27	100.0	0.0	0.0	0.0
	Squamous cell carcinoma of the penis	92	84	52.4	40.5	6.0	1.2
**Tumors of endocrine organs**	Adenoma of the thyroid gland	113	111	99.1	0.9	0.0	0.0
	Papillary thyroid carcinoma	391	363	97.0	2.8	0.0	0.3
	Follicular thyroid carcinoma	154	147	100.0	0.0	0.0	0.0
	Medullary thyroid carcinoma	111	109	100.0	0.0	0.0	0.0
	Parathyroid gland adenoma	43	34	100.0	0.0	0.0	0.0
	Anaplastic thyroid carcinoma	45	42	100.0	0.0	0.0	0.0
	Adrenal cortical adenoma	50	38	100.0	0.0	0.0	0.0
	Adrenal cortical carcinoma	28	28	100.0	0.0	0.0	0.0
	Phaeochromocytoma	50	50	100.0	0.0	0.0	0.0
	Appendix, neuroendocrine tumor (NET)	3	3	100.0	0.0	0.0	0.0
	Colorectal, neuroendocrine tumor (NET)	1	1	100.0	0.0	0.0	0.0
	Ileum, neuroendocrine tumor (NET)	4	4	100.0	0.0	0.0	0.0
	Lung, neuroendocrine tumor (NET)	10	9	100.0	0.0	0.0	0.0
	Pancreas, neuroendocrine tumor (NET)	49	49	93.9	4.1	2.0	0.0
	Colorectal, neuroendocrine carcinoma (NEC)	2	2	100.0	0.0	0.0	0.0
	Ileum, neuroendocrine carcinoma (NEC)	8	8	100.0	0.0	0.0	0.0
	Pancreas, neuroendocrine carcinoma (NEC)	3	3	100.0	0.0	0.0	0.0
**Tumors of haemotopoetic and lymphoid tissues**	Hodgkin Lymphoma	103	101	100.0	0.0	0.0	0.0
	Small lymphocytic lymphoma, B-cell type (B-SLL/B-CLL)	50	48	100.0	0.0	0.0	0.0
	Diffuse large B cell lymphoma (DLBCL)	113	108	100.0	0.0	0.0	0.0
	Follicular lymphoma	88	84	100.0	0.0	0.0	0.0
	T-cell Non Hodgkin lymphoma	25	25	100.0	0.0	0.0	0.0
	Mantle cell lymphoma	18	17	100.0	0.0	0.0	0.0
	Marginal zone lymphoma	16	15	100.0	0.0	0.0	0.0
	Diffuse large B-cell lymphoma (DLBCL) in the testis	16	16	100.0	0.0	0.0	0.0
	Burkitt lymphoma	5	4	100.0	0.0	0.0	0.0
**Tumors of soft tissue and bone**	Tenosynovial giant cell tumor	45	45	100.0	0.0	0.0	0.0
	Granular cell tumor	53	48	100.0	0.0	0.0	0.0
	Leiomyoma	50	50	100.0	0.0	0.0	0.0
	Leiomyosarcoma	94	93	100.0	0.0	0.0	0.0
	Liposarcoma	145	142	100.0	0.0	0.0	0.0
	Malignant peripheral nerve sheath tumor (MPNST)	15	15	100.0	0.0	0.0	0.0
	Myofibrosarcoma	26	26	100.0	0.0	0.0	0.0
	Angiosarcoma	74	68	100.0	0.0	0.0	0.0
	Angiomyolipoma	91	91	100.0	0.0	0.0	0.0
	Dermatofibrosarcoma protuberans	21	19	100.0	0.0	0.0	0.0
	Ganglioneuroma	14	13	100.0	0.0	0.0	0.0
	Kaposi sarcoma	8	6	100.0	0.0	0.0	0.0
	Neurofibroma	117	97	100.0	0.0	0.0	0.0
	Sarcoma, not otherwise specified (NOS)	74	72	100.0	0.0	0.0	0.0
	Paraganglioma	41	40	100.0	0.0	0.0	0.0
	Ewing sarcoma	23	19	100.0	0.0	0.0	0.0
	Rhabdomyosarcoma	7	7	100.0	0.0	0.0	0.0
	Schwannoma	122	112	100.0	0.0	0.0	0.0
	Synovial sarcoma	12	11	100.0	0.0	0.0	0.0
	Osteosarcoma	44	38	100.0	0.0	0.0	0.0
	Chondrosarcoma	40	29	100.0	0.0	0.0	0.0
	Rhabdoid tumor	5	5	100.0	0.0	0.0	0.0

**Table 2:** KLK7 immunostaining and tumor phenotype

				KLK7 immunostaining result				
			*n*	negative (%)	weak (%)	moderate (%)	strong (%)	*p*
Adenocarinoma of the colon and rectum	Tumor stage	pT1	88	95.5	4.5	0.0	0.0	0.6353
		pT2	442	93.7	6.1	0.2	0.0	
		pT3	1258	91.9	7.5	0.6	0.0	
		pT4	451	92.0	7.5	0.4	0.0	
	Grade	G1	4	100.0	0.0	0.0	0.0	0.8063
		G2	489	90.2	8.6	1.0	0.2	
		G3	55	87.3	12.7	0.0	0.0	
	Lmyph node status	pN0	1177	94.0	5.9	0.1	0.1	0.0005
		pN +	1057	90.4	8.7	0.9	0.0	
	Vascular infiltration status	V0	1611	92.8	7.0	0.1	0.1	0.0037
		V +	590	90.8	7.8	1.4	0.0	
	Lymphatic infiltration status	L0	739	95.5	4.3	0.0	0.1	< 0.0001
		L1	1474	90.6	8.6	0.7	0.0	
	Location	left	1265	94.3	5.5	0.2	0.0	0.0088
		right	459	90.0	9.4	0.7	0.0	
	Mismatch repair defective		88	92.0	5.7	2.3	0.0	
Liver cancer	Tumor stage	pT1	72	95.8	1.4	2.8	0.0	0.1118
		pT2	109	87.2	11.0	0.9	0.9	
		pT3-4	76	93.4	5.3	1.3	0.0	
	Lmyph node status	pN0	93	93.5	4.3	2.2	0.0	0.0382
		pN +	62	80.6	16.1	3.2	0.0	
	Grade	G 1	36	97.2	2.8	0.0	0.0	0.4041
		G 2	157	90.4	6.4	2.5	0.6	
		G 3	61	91.8	8.2	0.0	0.0	
Endometrioid endometrial carcinoma	Tumor stage	pT1	87	94.3	5.7	0.0	0.0	0.1207
		pT2	22	95.5	0.0	4.5	0.0	
		pT3-4	27	88.9	11.1	0.0	0.0	
	Lmyph node status	pN0	42	90.5	9.5	0.0	0.0	0.3566
		pN +	24	87.5	8.3	4.2	0.0	
Endometrioid carcinoma of the ovary	Tumor stage	pT1	24	83.3	16.7	0.0	0.0	0.3779
		pT2	4	75.0	25.0	0.0	0.0	
		pT3	5	100.0	0.0	0.0	0.0	
	Lmyph node status	pN0	19	84.2	15.8	0.0	0.0	0.6824
		pN1	9	77.8	22.2	0.0	0.0	
Serous carcinoma of the ovary	Tumor stage	pT1	36	38.9	52.8	8.3	0.0	0.5420
		pT2	43	44.2	48.8	4.7	2.3	
		pT3	261	49.4	40.2	9.6	0.8	
	Lmyph node status	pN0	83	44.6	44.6	9.6	1.2	0.4142
		pN1	163	47.2	46.0	6.7	0.0	
Pancreas adenocarcinoma	Tumor stage	pT1	15	73.3	26.7	0.0	0.0	0.2744
		pT2	72	63.9	29.2	6.9	0.0	
		pT3	398	61.8	29.9	6.8	1.5	
		pT4	31	74.2	12.9	12.9	0.0	
	Grade	1	17	70.6	23.5	0.0	5.9	0.3752
		2	366	62.3	29.8	7.4	0.5	
		3	109	64.2	27.5	6.4	1.8	
	Lmyph node status	pN0	114	63.2	29.8	5.3	1.8	0.7658
		pN +	401	63.3	28.2	7.5	1.0	
	Resection margin status	R0	261	67.0	26.4	5.7	0.8	0.2911
		R1	215	59.5	30.7	7.9	1.9	
	Mismatch repair status	proficient	464	64.0	28.9	6.3	0.9	0.3143
		deficient	4	100.0	0.0	0.0	0.0	
Papillary thyroid carcinoma								
	Tumor stage	pT1	140	98.6	1.4	0.0	0.0	0.0132
		pT2	75	100.0	0.0	0.0	0.0	
		pT3-4	98	91.8	7.1	0.0	1.0	
	Lmyph node status	pN0	85	95.3	4.7	0.0	0.0	0.2076
		pN +	120	98.3	1.7	0.0	0.0	
Stomach cancer	Histological type	diffuse	92	96.7	2.2	1.1	0.0	0.1035
		intestinal	88	94.3	5.7	0.0	0.0	
		mixed	60	88.3	11.7	0.0	0.0	
	Tumor stage	pT1-2	59	89.8	10.2	0.0	0.0	0.4286
		pT3	128	89.8	10.2	0.0	0.0	
		pT4	125	95.2	4.8	0.0	0.0	
	Lmyph node status	pN0	77	93.9	6.1	0.0	0.0	0.5413
		pN1	208	90.8	8.7	0.0	0.4	
	Mismatch repair status	proficient	255	90.2	8.6	0.8	0.4	0.8144
		deficient	40	90.0	10.0	0.0	0.0	
Squamous cell carcinoma of different sites*	Tumor stage	pT1	278	58.6	37.1	2.2	2.2	0.4833
		pT2	279	59.9	34.8	4.7	0.7	
		pT3	150	54.0	42.0	2.7	1.3	
		pT4	137	54.0	40.9	4.4	0.7	
	Lmyph node status	pN0	331	58.3	36.3	3.9	1.5	0.3838
		pN +	321	60.7	36.4	2.2	0.6	
	Grade	G1	34	44.1	38.2	14.7	2.9	< 0.0001
		G2	418	45.5	47.6	4.8	2.2	
		G3	267	70.0	28.5	1.1	0.4	

*Abbrevations*: *pT* pathological tumor stage, *pN* pathological lymph node status

*oral, pharynx, larynx, esophagus, lung, cervix, vagina, vulva, penis, skin, and anal canal

## Discussion

Our successful analysis of 12,345 tumors from 147 entities identified KLK7 expression in 64 cancer categories and enabled a ranking of tumor types according to their KLK7 positivity rate.

**Fig. 4:**
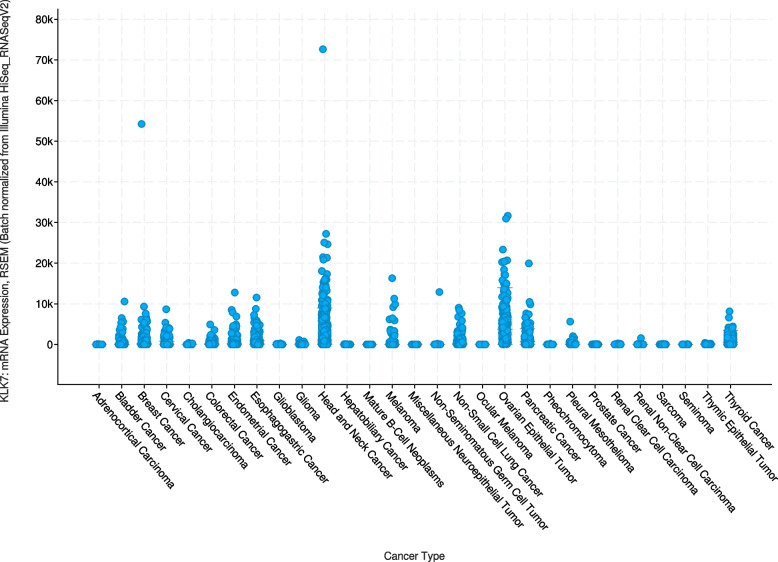
KLK7 in the TCGA data set and in the literature. KLK7 mRNA expression in different cancer types. Data plot generated from the cBioPortal [42, 43] database querying 10,976 samples from the “TCGA PanCancer Atlas Studies” sample set including 32 studies with KLK7 mRNA sequencing data

**Fig. 5:**
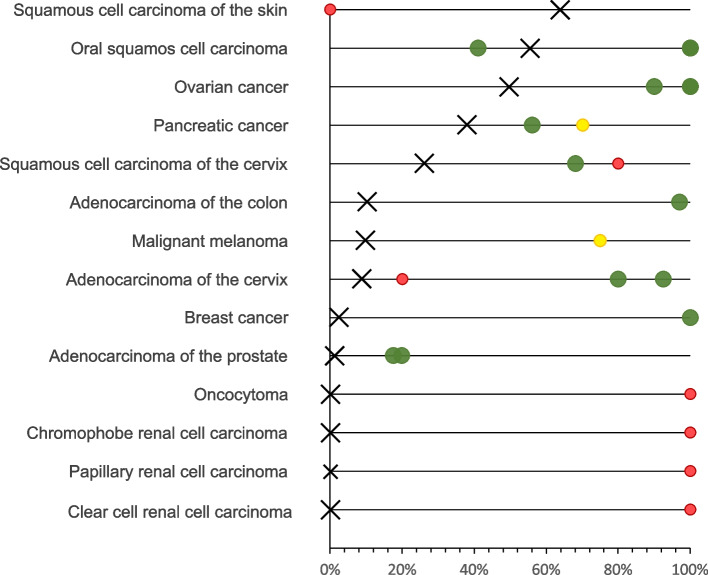
Comparison with previous KLK7 literature. Comparison of the percentage of samples with KLK7 expression (at least weak staining) with the corresponding data from published studies. An “X” indicates the fraction of KLK7 positive cancers in the present study, dots indicate the reported frequencies from the literature for comparison: red dots mark studies with ≤ 10 analyzed tumors, yellow dots mark studies with 11–25 analyzed tumors, and green dots mark studies with > 25 analyzed tumors. All studies are listed in the reference list

The most commonly KLK7 positive cancers included squamous cell carcinomas from various sites of origin, ovarian carcinomas, pancreatic adenocarcinomas, and salivary gland tumors. This is largely consistent with RNA expression data from The Cancer Genome Atlas Research Network (https://www.cancer.gov/tcga), suggesting highest rates and levels of KLK7 expression in cancers of the head and neck, the ovary and the pancreas (Fig. 4). Our data markedly expand the available data on KLK7 protein expression in cancer. Fifty of the 64 tumor categories with at least occasional KLK7 expression had not been studied by IHC before. Previous IHC studies had analyzed a total of 1,646 samples from 14 different tumor entities (summarized in Fig. 5) and often described substantially higher positivity rates than found in this study. For example, KLK7 positivity rates of ≥ 90% have been described for breast cancers [17], ovarian carcinomas [12–14], oral squamous cell carcinomas [20, 22, 25], adenocarcinomas of the colon [21], adenocarcinoma of the cervix [23], and different subtypes of renal cell carcinomas [27] in individual studies. Most of these discrepancies are likely to be caused by the use of different antibodies, staining protocols and definitions of thresholds to determine KLK7 positivity. Existing RNA data, along with our IHC results, do not suggest a high frequency of high KLK7 expression levels in these tumor entities [35–38]. Other reasons for discrepant findings may include variable expression in a specific molecular background. This might particularly apply for the breast cancers in our study. The general lack of detectable KLK7 expression fits well to earlier studies reporting downregulation of KLK7 mRNA in breast cancers [39, 40], but there are also studies suggesting that KLK7 can be upregulated specifically in hormone receptor negative [41] and triple negative breast cancers [17].

A frequent KLK7 expression in squamous cell carcinomas of various sites was expected based on the function of the protein in the desquamation of corneocytes. Desquamation of keratinized cells also occurs in cancers [44] and explains the expression of KLK7 in keratinized squamous cell carcinomas. Keratinization (or leucoplakia) is considered as a precancerous process in squamous epithelium [45–47] and defines a low histologic grade of malignancy in squamous cell carcinomas [48]. A significant association between KLK7 positivity and low grade could therefore be expected in these cancers. Cytokeratin 10, another protein that is specifically expressed in cornifying squamous epithelium, has also been found to be preferentially expressed in low grade squamous cell carcinomas [49]. In agreement with our observation, Leusink et al. found a link between strong KLK7 immunostaining and a favorable prognosis in a cohort of 83 squamous cell carcinomas of the oral cavity [26]. However, an association between high KLK7 protein levels and adverse pathological features or poor clinical outcome of squamous cell carcinomas has been described in two other studies on 30 and 80 cancers of the oral cavity [20, 25].

A poor prognosis of KLK7 expressing cancers could potentially be explained by in vitro and in vivo studies suggesting a relevant role of KLK7 in tumorigenesis, progression, and metastasis (summarized in [28]). The KLK7 zymogen is activated in the extracellular space to hydrolyze various extracellular matrix substrates (e.g. fibronectin 1/FN1 [50], thrombospondin 1/TSP1 [50]), cell membrane proteins (e.g. E-cadherin [11], desmoglein/DSG 1 and 2 [51]), secreted substrates (e.g. insulin growth factor binding protein/IGFBP 3 and 6 [50]), and other substrates (e.g. midkine [52], pro-matrix metalloprotease/MMP10 [50]). Studies suggested a direct or indirect effect of KLK7 on cell proliferation [19, 52], cell–cell adhesion [50, 51, 53], cell shedding [50], cell migration [50, 52, 54], cell invasion [11, 19, 52, 54], extracellular matrix organization [50, 55], EMT [11, 54], angiogenesis [28, 50], and metabolic reprogramming [28, 50] through the activation or inactivation of KLK7 substrates. For example, KLK7-mediated inactivation of E-cadherin – a hallmark of EMT [56]—and DSG1 and 2 promotes metastasis by inducing cell invasion and migration as well as reducing cell aggregation [11, 51]. KLK7-mediated inactivation of TSP1 may lead to cell proliferation, cell migration, cell invasion, and angiogenesis through the activation of the PI3K/Akt/mTOR pathway and VEGFR2 signaling [50, 57, 58]. KLK7-mediated inactivation of IGFBP3 and 6 may lead to cell proliferation through the activation of the PIK3/Akt, MEK/ERK, and Wnt/ß-Catenin pathways [50, 59] and changes in glycolysis, glycogen synthesis, and gluconeogenesis through the PI3K/Akt/mTOR pathway [50, 59, 60]. KLK7-mediated activation of proMMP10 and proMMP9 may leads to cell invasion and cell migration through the MMP-mediated hydrolysis of extracellular matrix and basement membrane components [50, 61, 62]. Furthermore, KLK7 overexpression resulted in a switch from a proliferative to an invasive phenotype in melanoma cells [19]. However, our own analyses of cancer entities with data on histomorphological features of malignancy did not provide evidence for a paramount prognostic impact of KLK7 expression on cancer malignancy. Only 7 of 28 analyses comparing KLK7 immunostaining and prognostic features resulted in significant associations. Because the number of statistical analyses was so large in our study (*n* = 28) that a statistical adjustment (Bonferroni correction: *p* ≤ 0.0018) invalidated almost all significant results, it was conspicuous that 4 of the 7 significant data involved nodal metastasis or tumor infiltration into blood or lymph vessels. These observations would indeed be consistent with a role of KLK7 associated cell dissociation in the process of metastasis.

It is an advantage of our study that only a single 0.6 mm tissue core was analyzed per cancer. The one punch per cancer approach allows for maximal standardization of the analysis because the same amount of tissue (approx. 0.28 mm^2^) is studied from each tumor. In contrast, the use of multiple punches per tumor almost inevitably leads to statistical bias because virtually never all samples from each tumor are interpretable. Others and we have earlier shown that one 0.6 mm core per cancer is sufficient to find clinically relevant associations between molecular markers and tumor phenotype (reviewed in [63]). For example, Torhorst et al. [64] analyzed 1–4 TMA cores each for p53, ER and PR and demonstrated that known associations between these markers and patient prognosis can be found irrespective of whether four tissue cores per tumor were analyzed separately, or a combined result was generated from the four cores.

As KLK7 is only expressed in a limited number of normal tissues and de-novo expression can occur in cancer, KLK7 may represent a suitable drug target for the treatment of cancer. Several studies have indeed demonstrated successful KLK7 inhibition by protein inhibitors and small molecule inhibitors in preclinical studies (summarized in [28]). In addition, some studies have already shown an antitumor effect of KLK7 inhibition. For example, small molecules inhibiting KLK7 have efficiently inhibited the proliferation, migration and invasion of pancreatic cancer cells [65], and cell shedding of plantar stratum corneum cells [6]. Our data suggest that ovarian, endometrial, pancreatic, and gallbladder carcinomas might represent suitable tumor entities for clinical trials once anti-KLK7 drugs will move towards testing in human patients.

Considering the large scale of our study, our assay was extensively validated by comparing our IHC findings in normal tissues with data obtained by another independent anti-KLK7 antibody and RNA data derived from three different publicly accessible databases [35–38]. To ensure an as broad as possible range of proteins to be tested for a possible cross-reactivity, 76 different normal tissues categories were included in this analysis. Validity of our assay was supported by the detection of significant KLK7 immunostaining in organs with documented KLK7 RNA expression (esophagus, salivary gland, kidney, vagina, cervix, fallopian tube, tonsil, and the skin) with the only exception of breast tissue which always stained KLK7 negative both on TMAs and on large sections. As KLK7 RNA was not detected in the FANTOM5 dataset [35, 36], it appears possible that some breast samples from other databases were contaminated by KLK7 RNAs derived from the skin covering breast tissues. KLK7 positivity in a fraction of cells in corpuscles of Hassall’s was the only IHC finding which was not matched by RNA data. This staining is conceivable, however, because of the squamous epithelial nature of corpuscles of Hassall’s. As for all other normal tissue results, KLK7 positivity of corpuscles of Hassall’s was confirmed by the use of a second independent antibody. In the thymus, the KLK7 positive cells constitute such a small fraction of the total number of cells that KLK7 RNA may not occur at detectable quantities in RNAs extracted from whole organ tissue samples. Independence of the two KLK7 antibodies used in this study is documented by cytoplasmic smooth muscle staining and nuclear staining in several organs (often predominating on nuclear membranes) which was seen by EPR22594-203 but not by MSVA-707M.

## Conclusion

Our data provide a comprehensive overview on KLK7 expression in normal and neoplastic human tissues. KLK7 expression predominates in squamous cell carcinomas of different organs but is also common in ovarian and biliopancreatic adenocarcinomas as well as adenocarcinomas of the upper gastrointestinal tract. The prognostic relevance of KLK7 expression and the possible role of KLK7 as a drug target needs to be further investigated.

### Supplementary Information

Supplementary Material 1.

Supplementary Material 2.

Supplementary Material 3.

## Data Availability

All data generated or analyzed during this study are included in this published article.

## Abbreviations

IHC: Immunohistochemistry
KLK7: Kallikrein-related peptidase 7
pT: Pathological tumor stage
pN: Pathological lymph node status
TMA: Tissue microarray
