# Supplementary material for: KLK7 expression in human tumors: a tissue microarray study on 13,447 tumors
Source: BMC Cancer. 2024 Jul 3;24:794. doi: 10.1186/s12885-024-12552-8 (PMC11221178; doi:10.1186/s12885-024-12552-8)
Supplement: Supplementary file 1 — Supplementary Material 1. [file 12885_2024_12552_MOESM1_ESM.pdf]

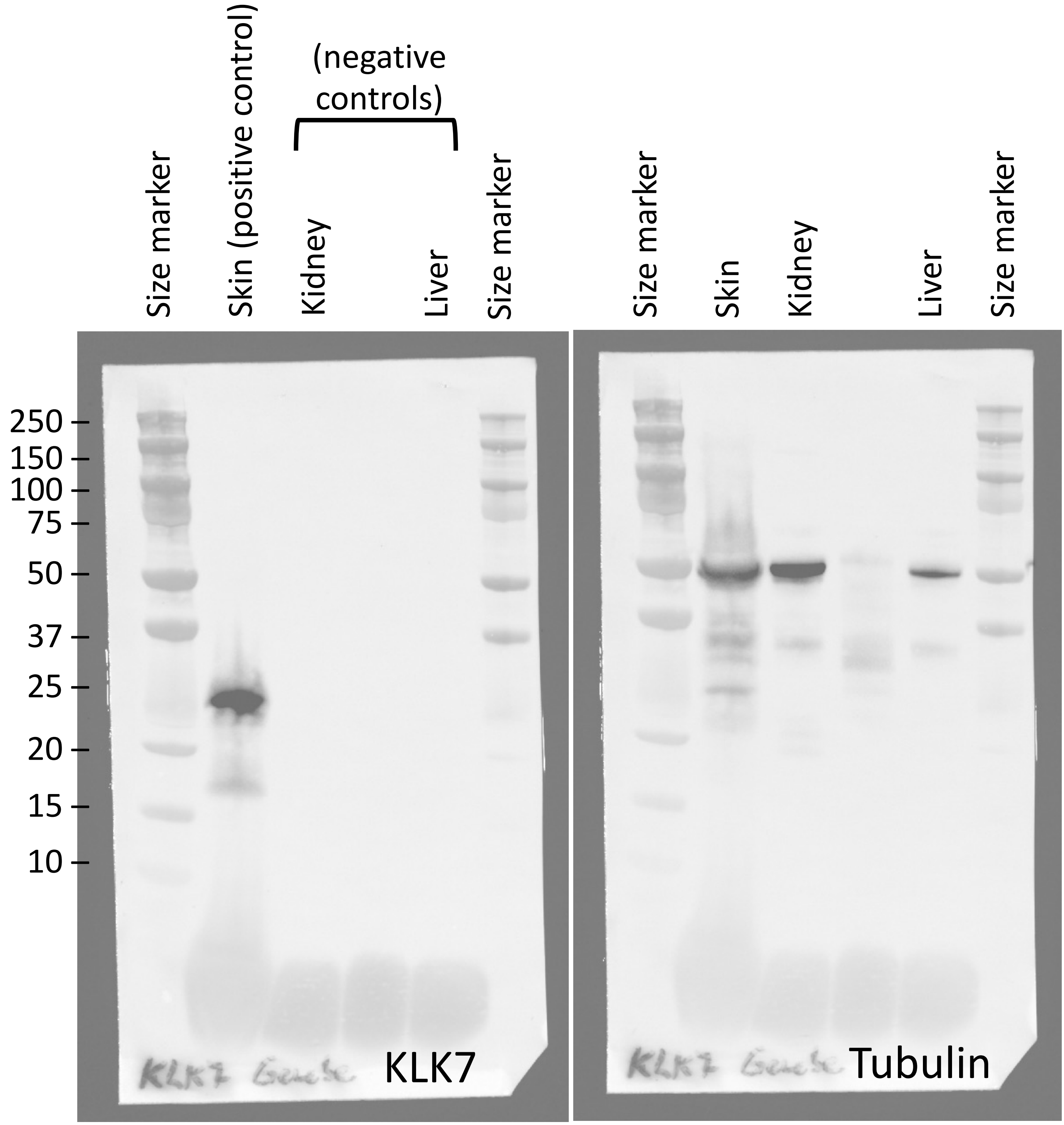

Supplementary Figure 1: Left: Western blot analysis of antibody MSVA-707M in normal skin, kidney, and liver tissues. The size of the protein bands detected by the antibody correspond to KLK7 isoforms 1 (uniprot ID P49862-1, 27.5 KDa) and 2 (uniprot ID P49862-2, 19.8 KDa). **Right:** Western blot analysis of anti-tubulin as a loading control of the same blot. The lane between kidney and liver did not contain sufficient protein (lack of a tubulin band) and was excluded from the analysis.
