# Supplementary material for: KLK7 expression in human tumors: a tissue microarray study on 13,447 tumors
Source: BMC Cancer. 2024 Jul 3;24:794. doi: 10.1186/s12885-024-12552-8 (PMC11221178; doi:10.1186/s12885-024-12552-8)
Supplement: Supplementary file 2 — Supplementary Material 2. [file 12885_2024_12552_MOESM2_ESM.pdf]

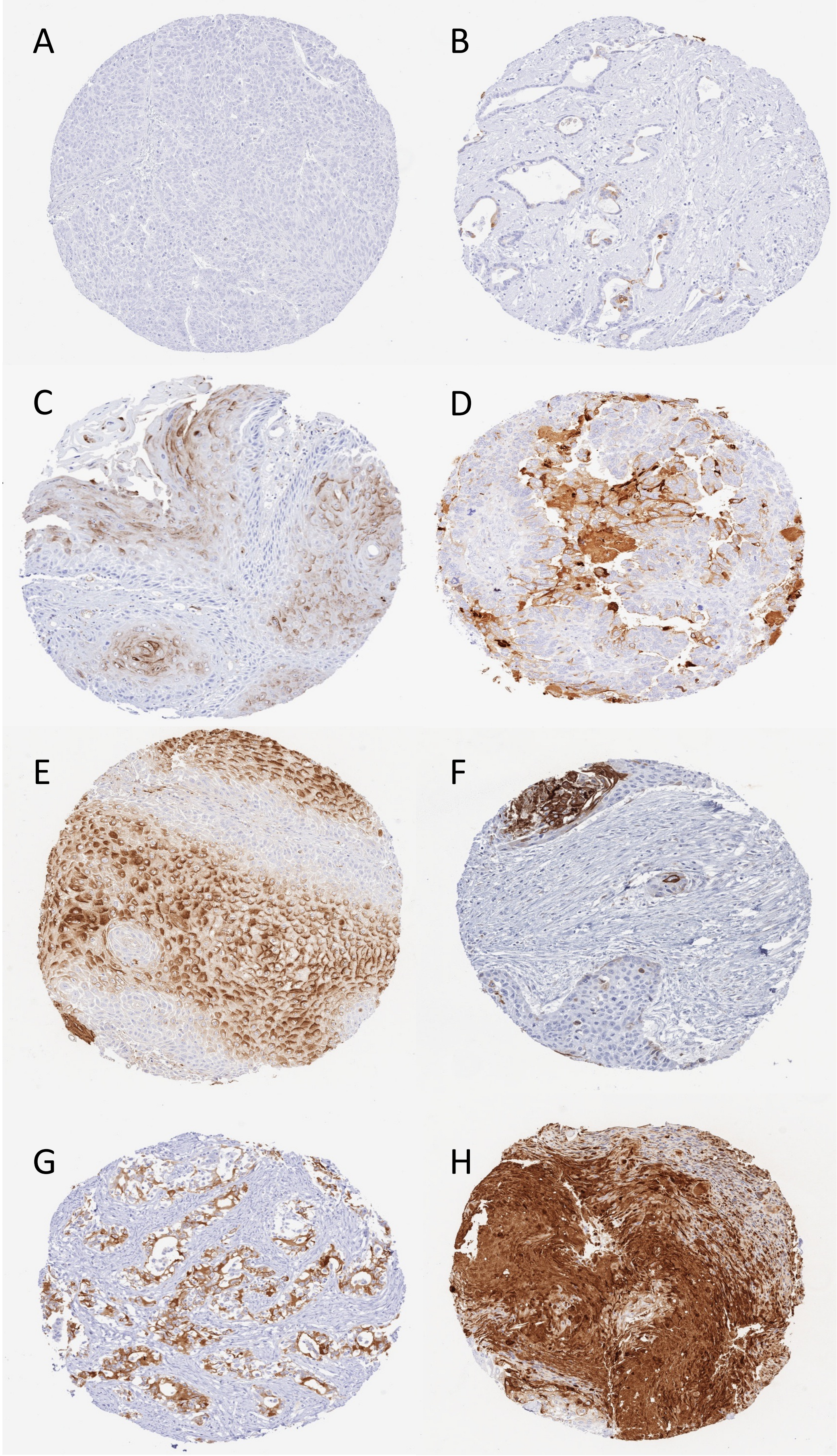

Supplementary Figure 2: Examples of KLK7 immunostaining and corresponding scores. **A)** Squamous cell carcinoma of the penis, staining intensity (int) 0 = negative; **B)** Gastric adenocarcinoma, intestinal type, int 1+ in 30% of tumor cells = weak, **C)** Squamous cell carcinoma of the anal canal, int 1+ in 60% of tumor cells = weak; **D)** Squamous cell carcinoma of the skin, int 2+ in 10% of tumors cells = weak, **E)** Squamous cell carcinoma of the anal canal, int 2+ in 70% of tumor cells = moderate; **F)** Squamous cell carcinoma of the vulva, int 3+ in 30% of tumors cells = moderate; **G)** Serous carcinoma of the ovary, int 2+ in 100% of tumor cells = strong; **H)** Squamous cell carcinoma of the skin, int 3+ in 100% of tumor cells = strong.
