# Supplementary material for: KLK7 expression in human tumors: a tissue microarray study on 13,447 tumors
Source: BMC Cancer. 2024 Jul 3;24:794. doi: 10.1186/s12885-024-12552-8 (PMC11221178; doi:10.1186/s12885-024-12552-8)
Supplement: Supplementary file 3 — Supplementary Material 3. [file 12885_2024_12552_MOESM3_ESM.pdf]

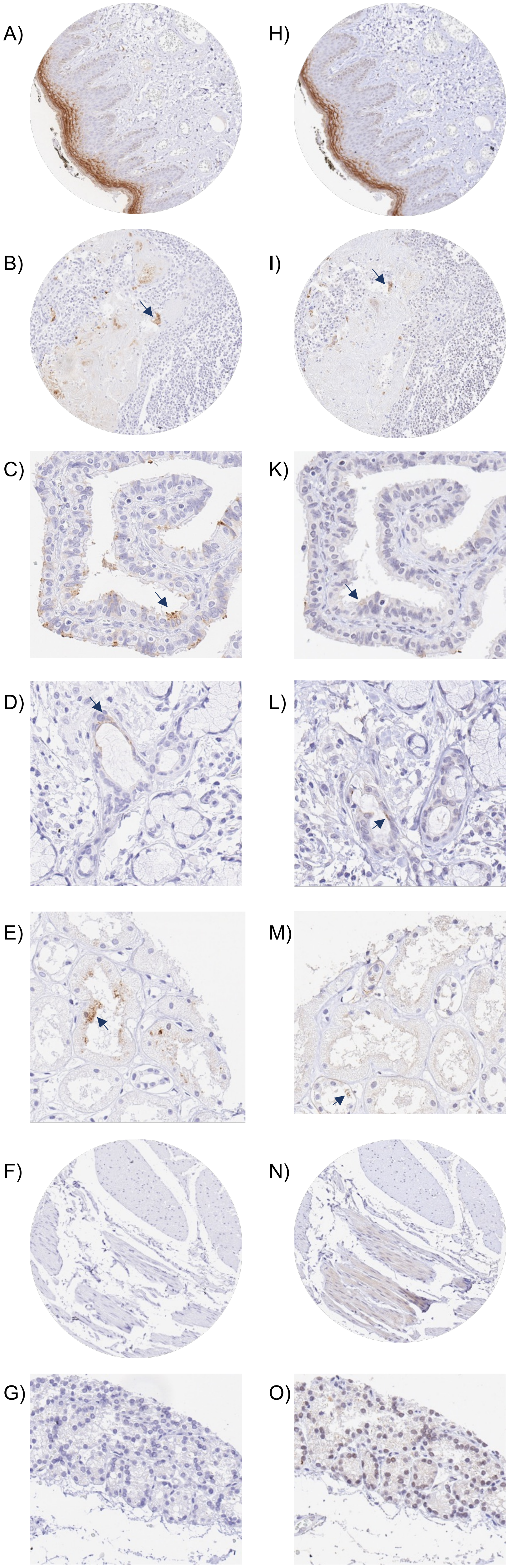

Supplementary Figure 3. Immunohistochemistry validation by comparison of antibodies. Using MSVA-707M, a predominantly membranous KLK7 positivity is seen in the granular cell layer of the anal skin **(A)**, few squamous epithelial cells in the tonsil crypt **(B)**, few individual cells in the fallopian tube **(C)**, few epithelial cells in a submandibular gland **(D)**, and in few tubular cells of the kidney **(E)** while KLK7 staining was absent in the muscular wall of the colon **(F)** and in the parathyroid **(G)**. Using clone EPR22594-203, a weaker but otherwise similar KLK7 positivity is seen in the anal skin **(H)**, tonsil crypts **(I)**, fallopian tube **(K)**, submandibular gland **(L)**, and in the kidney **(M)**. Clone EPR22594-203 generally resulted in more background staining and tended towards cytoplasmic staining in smooth muscle cells such as in the colon wall **(N)** and occasional nuclear staining such as in the parathyroid **(O)**. The images **A-G** and **H-O** are from consecutive tissue sections. Relevant areas are highlighted by arrows in some images.
